# A wireless sensor system for a biofeedback training of hammer throwers

**DOI:** 10.1186/s40064-016-3069-5

**Published:** 2016-08-22

**Authors:** Ye Wang, Bingjun Wan, Hua Li, Gongbing Shan

**Affiliations:** 1 Department of Mathematics and Computer Science, University of Lethbridge, Lethbridge, AB Canada; 2 School of Physical Education, Shaanxi Normal University, Xi’an, China; 3 Biomechanics Lab, Faculty of Arts and Science, University of Lethbridge, 4401 University Drive, Lethbridge, AB T1K 3M4 Canada; 4 Department of Physical Education, Xinzhou Teachers’ University, Xinzhou, Shanxi China

**Keywords:** Wireless sensor networks, Hammer-throw, Arduino, XBee

## Abstract

Hammer-throw has a long-standing history in track and field, but unlike some other sports events, men’s hammer throw has not seen a new world record since 1986. One of the possible reasons for this stagnation could be the lack of real-time biomechanical feedback training. In this study, we proposed to establish scientifically described training targets and routes, which in turn required tools that could measure and quantify characteristics of an effective hammer-throw. Towards this goal, we have developed a real-time biomechanical feedback device—a wireless sensor system—to help the training of hammer-throw. The system includes two sensors—an infrared proximity sensor for tracing the hip vertical movement and a load cell for recording the wire tension during a hammer-throw. The system uses XBees for data transmission and an Arduino processor for data processing and system control. The results revealed that the wire tension measurement could supply sufficient key features for coaches to analyze hammer-throw and give real-time feedback for improving training efficiency.

## Background

Effective human motor skill learning/training not only benefits athletes but can also promote more active lifestyles in the general population (Chen and Ennis 2004; Li et al. 2016; Wan and Shan 2016). The two key components in motor learning are practice and biofeedback (Schmidt and Lee 2011). Previous studies have shown that, when properly understood and applied, biofeedback can strongly enhance the practice of human motor skills (Shan et al. 2004; Visentin et al. 2008). Generally, there are three types of biofeedback: physiological (e.g. heart rate), neurological (e.g. EEG/brain-wave), and biomechanical (e.g. joint angles and force applied) (Tate and Milner 2010). While physiological and neurological feedback devices are commonly seen in practice, biomechanical feedback devices are still in their developing phase. The reasons for the current situation could be the following points: (1) effective biomechanical feedback should relate to the invisible forces (i.e. we can only feel the effect of a force, but cannot see it; the only way for its visualization/quantification is through a force measurement device, such as a scale) controlling the limb movement of human motor skills (Shan and Westerhoff 2005), and (2) motor skill learning and optimization must be tailored to an individual body structure and the activity being examined (Shan and Bohn 2003; Shan et al. 2015; Visentin et al. 2015). Because of the advance of wearable/wireless sensor systems, a “tailored” biomechanical feedback device would be theoretically possible for individualized training. Such a device would allow the users to self-correct problems based on such biofeedback (such as the invisible forces during a movement) provided by feedback devices.

One of the possible tools for biomechanical feedback training is wireless sensor networks (WSNs). WSNs are composed of distributed nodes connected with sensors which communicate with each other and send/receive data to the base station. Each sensor node has a battery for power, a microprocessor for programming, and a transceiver for communication. In this paper, the proposed architecture is based on Xbee/Arduino modules where Xbee is used for communication and Arduino used to control and process the data. WSNs have been applied in a wide range of applications, such as in agriculture (Keshtgari and Deljoo 2012), in health care monitoring (Mansor et al. 2013), in smart home technology (Lu et al. 2014), in environment observation (Lazarescu 2012) and ecosystem (Du et al. 2015). The successes of WSNs in the above areas suggest that their application potential in human motor learning and training is high.

One of the practical challenges for establishing the biomechanical feedback device is its size, not only the sensor size but also the microprocessor unit. It should be tiny and wearable, but at the same time, it should not constrain an athlete’s movement. IEEE 802.15.4-compliant (Digi 2014a) transceivers are typically used for communications in WSNs. The performance of smart grid applications with IEEE 802.15.4-compliant transceivers has been studied by Bilgin and Gungor (2012). Piyare and Lee (2013) analyzed the efficiency of XBee ZB module-based WSNs regarding the received signal strength indication (RSSI), delay, throughput, energy, etc.

Several successful application examples using the above technology have been reported. They range from collecting climatologic data (Keshtgari and Deljoo 2012), measuring body temperature development and heart rate in patients (Mansor et al. 2013; Kioumars and Tang 2011), and monitoring radioactive materials (Ding et al. 2009). These applications share the communication protocols (ZigBee) and RF hardware, but not necessarily the underlying computing platform.

In these examples, the sensors are relatively static, and on the receiver side, the computer is connected to another XBee module to make the wireless communication available, and the computer is used to monitor and process the data. However, for a biofeedback application in human movement, further development is required.

As discussed above, biomechanical feedback must be tailored to a specific activity. In the current study, the hammer throw is chosen for the development of the biomechanical feedback device. Hammer throw has a long-standing history in track and field, but unlike some other events, hammer throw has not seen a new world record since 1986 (IAAF 2015). One of the possible reasons for this stagnation could be the lack of scientific feedback data needed for the training. While extensive 3D motion analysis technologies do exist for hammer throw, practitioners have reported that they are too cumbersome to be useful for training (Shan et al. 2012). The main issue is the time-consuming data collection and processing. Such a procedure would make biomechanical feedback available for practitioners after weeks, reducing the practicality of 3D motion caption in hammer throw training. Due to the complexity of the movement and the difficulty of its data collection, hardly any scientific research exists for the hammer throw. Therefore, a real-time biofeedback tool, e.g. a wireless wearable sensor system, is desired in practice.

**Fig. 1:**
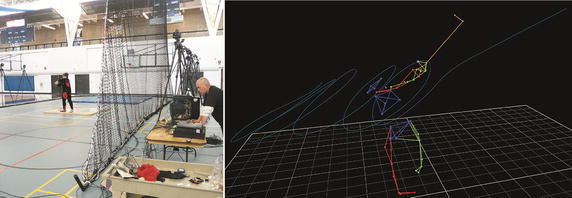
3D motion analysis of hammer throw. *Left* The set-up of the 3D motion capture with 12 high-speed cameras (VICON MX40, 250 Hz); *right* 3D reconstruction of a throw

Through a pilot study (Fig. 1), we found that wire tension and vertical hip displacement measurements might be sufficient to substitute 3D motion capture when analyzing the hammer throw. As such, one could assume that the hammer throw could be improved by a real-time biofeedback of tension development during a throw. Since the kinetic energy generated by an athlete’s turning is finally transferred to the hammer, quantifying the tension would be highly linked to the motor skill control and could supply information guiding the optimization of the throw.

Therefore, the current study aimed to develop a new, wearable real-time biomechanical feedback device, which would measure (1) real-time wire tension and (2) vertical hip displacement. Specifically, the hardware development aimed to prototype a wireless data collection unit and the software development intended to equip the feedback device with software that can collect wire tension and vertical hip displacement measurements, receive and store tension and displacement data, perform primary data processing functions and include a graphical user interface for real-time data visualization. Such a feedback system would have further potentials for development to (1) establish how to reach desirable tension and displacement during a throw, and ultimately (2) provide biomechanically-guided training plans customized to each athlete’s anthropometrical data. In short, the system developed would have great potential to be both a research tool for better understanding of hammer throw movements and a user-friendly training tool for coaches and athletes.

## Results and discussions

The aims of the study were to develop a wearable biomechanical feedback device. We have successfully prototyped the device. The applicability of the device was tested in the training sessions of the Canadian hammer throw team. The Human Subjects Research Committee of the University of Lethbridge/Canada scrutinized and approved the protocols as meeting the criteria of ethical conduct for research involving humans. The athletes were informed that the device would be used for collecting data related to their throws. They signed an approved consent form and voluntarily participated in the data collection.

**Fig. 2:**
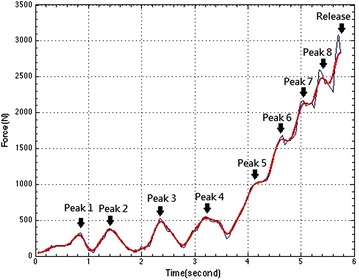
Typical tension excursion found in Canadian Champion’s throws. His throw was 62 m

**Fig. 3:**
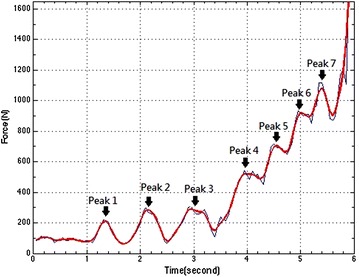
Typical tension excursion found in the female subject’s throws. Her throw was 39 m

Figures below show the tension development of a male (Fig. 2) and a female (Fig. 3) athlete during his/her throws. The blue curves in the figures were drawn from the raw data, while the red curves were the results of the filtered data (Butterworth filter).

The male data is from the Canadian Champion’s throw. His best performance, achieved in May 2008 in Lethbridge, Alberta, still stands as the current Canadian record. In Fig. 2, one can see that there are several peaks before reaching the maximum release point. The video capture showed that the throw could be divided into two phases: (1) preparation—the subject pulled up the hammer from the ground and then, swung the hammer for two circles before starting the next phase; (2) body turning—the subject performed four and a half turns before the release of the hammer. The first three peaks represented the preparation phase—Peak 1: pull-up, Peak 2 and 3: two circles of hammer swing. The preparation ended at 2.8 s, and then the athlete entered the turning phase.

The video data unveiled that the turning consisted of double-support and single-support. Double-support is the duration of each turn where both feet are in contact with the ground. Conversely, single-support is the portion of each turn where the right foot (for a right-handed thrower) is in the air while the left foot remains in contact with the ground. Based on physics, a thrower needs to extend double-support as much as possible because the only way to increase speed is to drive or push with the right foot and the right foot is only on the ground during the double-support, so this is truly the only time to accelerate body rotation. By lengthening the double-support time (while shortening the single-support time), one can push effectively, resulting in increasing the angular velocity of the body-turning thus increasing the speed of the ball. Therefore, it can increase the length of the flight.

During each turn, there was acceleration (uphill part of a peak) and deceleration (downhill part of a peak). From a physics point of view, the first turn (start of body rotation) and the last half turn (release of the hammer) should be different from the 2nd to 4th turns. The subsequent turns (2nd to 4th turns) should be identical in form with each turn gaining rotary speed on the previous turn. The tension measurement did show these characteristics. The data revealed that the first turning (Peak 4 in Fig. 2) was the slowest one with the longest single-support (longest downhill). The 2nd, 3rd and 4th turn (Peak 5, 6 and 7 in Fig. 2) were physically alike: longer acceleration (i.e. double-support) and shorter deceleration (single-support). They were faster than the 1st turn. The last half turn (double support, Peak 8 in Fig. 2) and the finishing body upward motion, i.e. both knee fast extension and trunk fast over-extension right before the athlete released the hammer (the increase after Peak 8 in Fig. 2) represented the final acceleration for maximizing the release speed of the ball. The slightly drop between the last half turning and the finishing body upward motion may indicate a loss of power. Improving the timely coordination between the two segmental controls could increase his performance. Collectively, the data indicated that the athlete rotated faster and faster, as the tension increased a portion per turn, and reached the maximum when he released the hammer. The video data proved that his rotation speed increased from turn to turn and the final body upward motion. Additionally, the quantification of ball speed based on Physics (Eq. 1) also confirmed the above motion characteristics (Table 1).

1 $$F = \frac{{m \times V^{2} }}{r},$$

where F is the wire tension, V is the ball speed, m the mass of the hammer and r is the radius (subject’s arm length plus the wire length) which was measured as 1.945 m for the male subject and 1.765 m for the female one. It should be mentioned that the male standard hammer weighs 7.257 kg and has a wire length 121.5 cm, while the female one weighs 4 kg and has a wire length 119.5 cm (IAAF 2015).

**Table 1:** The results of rotary speed in each turn and the release speed from the Canadian champion’s field tests

	Wire tension (N)	Max rotary speed in each turn (m/s)	Increase of rotary speed in each turn (%)
Turn 1 (Peak 4)	542.63	12.06	
Turn 2 (Peak 5)	1037.5	16.68	38.3
Turn 3 (Peak 6)	1632.22	20.92	25.4
Turn 4 (Peak 7)	2154.6	24.03	14.9
The release (Max)	2843.37	27.61	14.9

The field-test results of the female athlete are shown in Fig. 3 and Table 2. Although the video data showed that the male and female athlete performed a highly similar movement, i.e. pull-up, two circles of hammer swing and four and a half body turns, several differences could be clearly identified. First, the female maximum tension at the release reached only 57.4 % of that of the male athlete (1630.9 vs. 2843.37 N). Except for the gender difference in the muscle power, the weight of the ball plays a role (4 vs. 7.257 kg). Second, the female tension had one peak less than that of the male one. Third, there was a sudden tension increase during the last half turn. The above two characteristics could suggest that the segmental coordination/sequential segmental control was optimized by the female athlete, i.e. no power loss (or smooth connection) between the half turn and the finishing body upward motion (i.e. the release). Last, the maximum tension observed in the 1st turn was about the same for both athletes. The results indicated that they employed totally different motor control strategies, even though the videos showed very similar movements. A detailed comparison between Tables 1 and 2 reveals that the female athlete accelerated the ball faster that the male athlete during the preparation and the 1st turn (~3 m/s faster). Then the increase of her rotary speed was notably lower than that of his from the 2nd to the 4th turns, i.e. 19.3 versus 38.3, 14.2 versus 25.4 and 9.3 versus 14.9 % for 2nd, 3rd, and 4th turn respectively. Finally, her smooth transfer of the momentum from segments to the ball helped her gain more increase of the ball release speed than the male athlete.

**Table 2:** The results of rotary speed in each turn and the release speed from a female subject’s field tests

	Wire tension (N)	Max rotary speed in each turn (m/s)	Increase of rotary speed in each turn (%)
Turn 1 (Peak 4)	503.56	14.91	
Turn 2 (Peak 5)	716.27	17.78	19.3
Turn 3 (Peak 6)	933.32	20.29	14.2
Turn 4 (Peak 7)	1115.64	22.19	9.3
The release (Max)	1630.9	26.83	20.9

Which control strategy would be more effective? What is the optimized control for hammer throw? Should males and females use a different throw technique? Should the control pattern be individualized according to one’s physical condition? The results suggest more questions than supply solutions for the questions. Definitely, more applied studies using biofeedback device in training are needed to answer the questions. However, the current study implies that coaches could use the real time, biomechanical feedback tool to experiment various/possible motor control strategies for skill optimization in practice.

Inevitably, there are flaws in the prototyping. The current optical distance sensor requires that the sensor points vertically towards the ground to get the correct distance, i.e. the up-and-down movement of the hip. During the field tests, it was found that hip-orientation changed continuously during the turns; as such, the wireless distance sensor could not supply valid data. Further studies using alternative distance sensors are needed to investigate the hip movement to remedy the flaw for adding hip control into feedback in learning and motor skill optimization. Additionally, it is planned to use Bluetooth technology to implement the receiver node in a cell phone or a mobile device, such as iPhone, iPad or other tablets. In this case, coaches will have a more convenient way to perform the real-time biofeedback training. Finally, an improvement on the current structure or designing a PCB (Printed Circuit Board) is planned to minimize the device for more convenience to the athletes.

## Conclusions

Wireless sensor networks have great application potential in human motor skill learning and optimization. Using the example of the hammer throw, we have shown in the current study that properly designed WSN device could supply invisible control information of professional athletes. Such valuable information would help coaches establish real-time biofeedback training and improve the performance of athletes. Most importantly, the current study extends the WSNs application to a new area—the professional athletes training.

## Methods

**Fig. 4:**
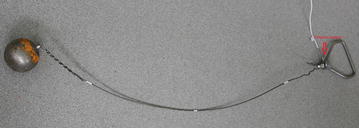
The prototype of biomechanical feedback device for hammer throw training

**Fig. 5:**
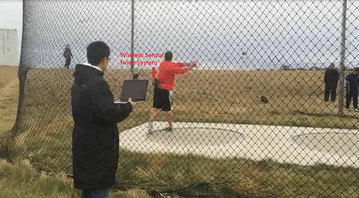
Field test

We have successfully prototyped the real-time biomechanical feedback device (Fig. 4) for hammer throw analysis and applied the device into training sessions of Canadian hammer throw team (Fig. 5). The followings are a detailed description of the methodology, including (1) hardware and system configuration, (2) programming and interface and (3) system calibration during our prototyping process.

### Hardware and system configuration

#### System architecture

The basic idea in our research is to establish a system of the WSN to receive two kinds of data: the distance from the athlete’s waist (hip) to the ground—up and down movement of the upper body—and the tension during the process of the hammer throw. In this system, we use a sensor node to collect data and send the data to a receiver node via wireless communication.

**Fig. 6:**
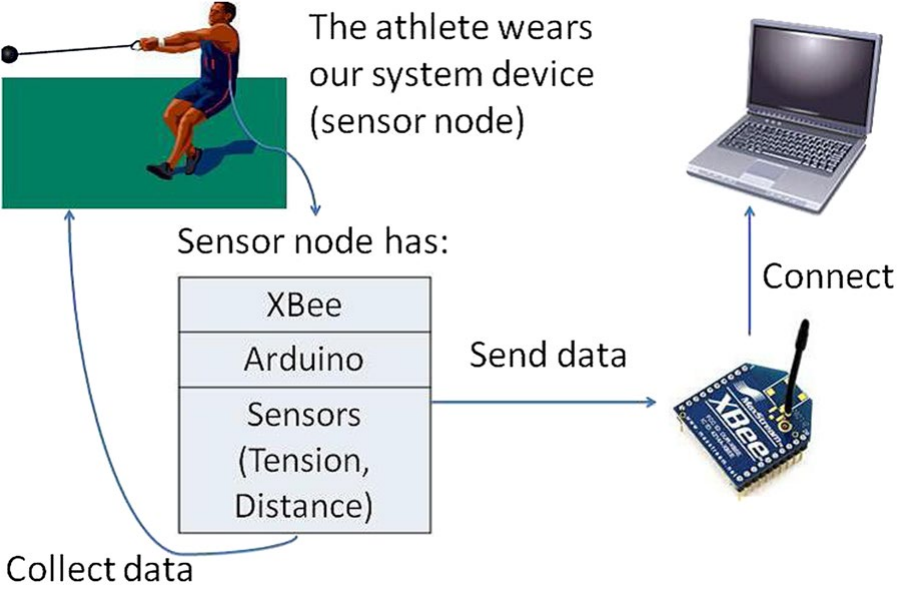
The architecture of the biofeedback system

**Fig. 7:**
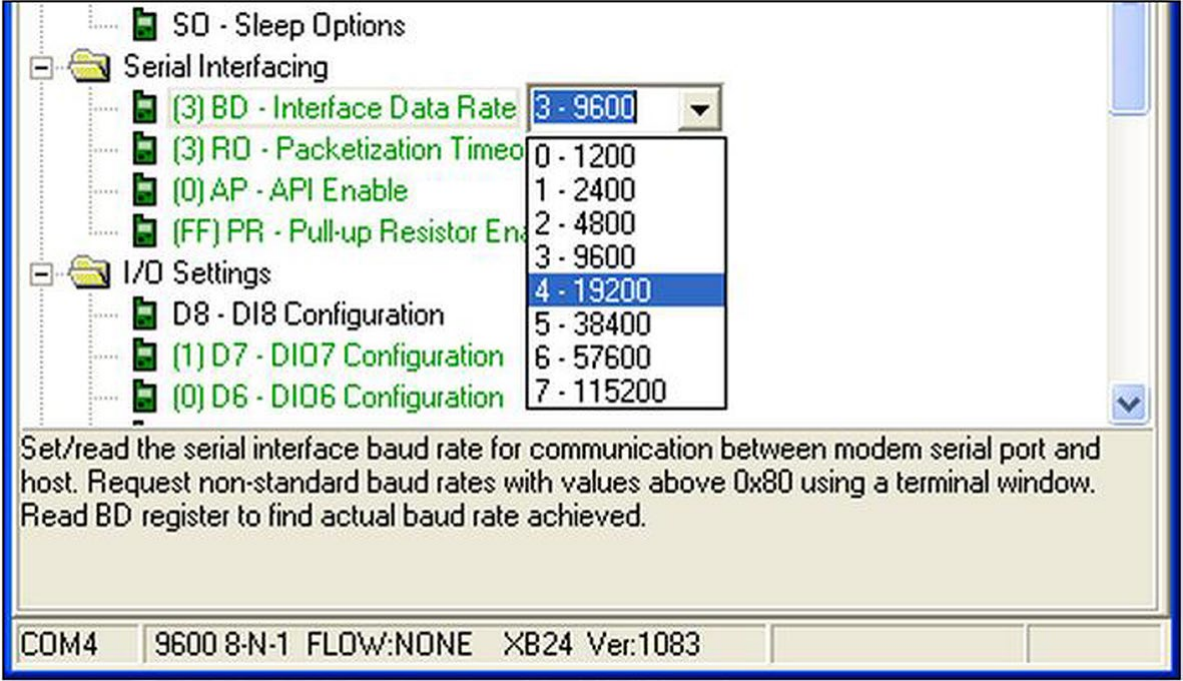
Baud rate change in XCTU

Figure 6 shows the architecture of our WSN system. We can tie our system device to the athlete’s waist. This device is the sensor node which is used for collecting data and sending data to the receiver node. We have a laptop for receiving and processing the data that is transferred from the sensor node. The critical part is a communication between these two nodes. We use one XBee for each of the nodes as a wireless transfer method. We used XCTU to configure the XBees in advance to make sure that they only recognize and communicate with each other. XCTU (Digi 2014b) is a free software application which is used to configure and test XBee RF modules through an easy graphical interface. We changed the baud rate, which indicates how fast the data can be sent or received on a serial line, from the default value (9600) to 57,600 to get a faster transmission speed. In Fig. 7 we can see that “BD—Interface Data Rate” which indicates the baud rate, in the menu of “serial interfacing”, can be changed. The range that we can change is from 1200 to 115,200, and there are eight levels (0–7). Every next level is twice as fast as the previous level. After setting the baud rate, we checked the other properties to make sure all the properties of our two XBees match with each other exactly. We used the “Write” button under the menu of “Modem Parameters and Firmware” to save any changes of our XBees.

#### Sensor node

**Fig. 8:**
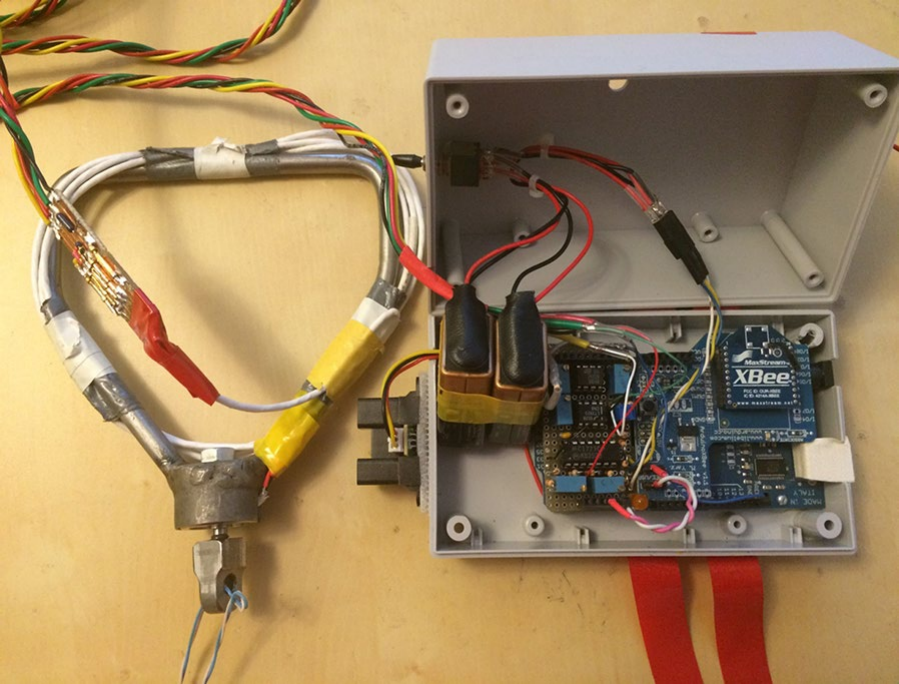
Sensor node. *Left* Tension sensor built into the handle; *right* distance sensor (attached to the *box*) and the microcontroller (in the *box*)

We have implemented the hardware of the sensor node which is shown in Fig. 8. The Arduino Mega (ATmega1280) board is used as a microcontroller. An XBee module is connected to the Arduino board for the wireless communication. There are two sensors in the sensor node. The distance sensor occupies the analog input pin 8 on the Arduino board, and the tension sensor occupies the analog input pin 9. The distance sensor shares power together with the Arduino microcontroller. We use two 9 V batteries to supply power to the tension sensor. Besides, we set a ten voltages regulator to provide the operating voltages for the load cell (tension sensor). It must be very precise for the load cell to provide the proper transfer function so that the output of the load cell is within its calibrated limits according to its calibration sheet. We include an amplifier which can bring the up signals from the load cell to generate the exact ten voltages as it requires. It allows us to avoid collecting the wrong data values in the case that the batteries cannot supply the load cell with the correct operating voltages. We also install an external SRAM (static random access memory) component onto the Arduino board to make sure we have enough memory to collect data. It turns out that the real-time data collection is fast enough to transfer all of the data in real time, so the extra memory is not used actually.

Figure 6 shows the hardware. It contains three basic components: two sensors, an Arduino board, and an XBee. There are two sensors in this system. One is for measuring the distance from the waist of the athlete to the ground. We use the infrared proximity sensor made by Sharp, which has an analog output varying from 2.8 V at 15 cm to 0.4 V at 150 cm (Sparkfun 2014). The other one is a load cell (tension sensor) for testing the wire tension during hammer throw. The load cell is produced by Omegadyne, and we use the type of LCFD-1 K, which can measure as high as 5000 N (Omegadyne 2014).

**Fig. 9:**
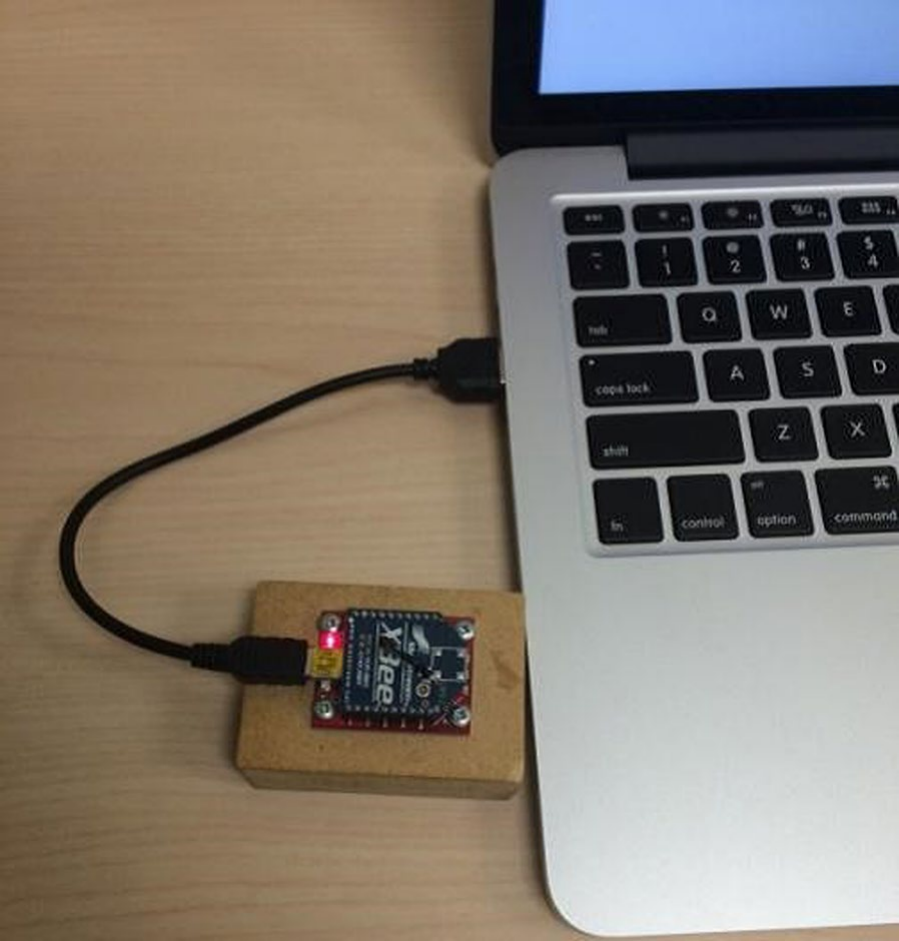
Receiver node

We use Arduino Mega (ATmega1280) board as the microcontroller of our system device. It has sixteen analog inputs (Analog In pin 0 to pin 15) (Arduino 2014a). We use two pins (A8 and A9) of those. The clock speed of Arduino is 16 MHz. According to the datasheet of Arduino Mega, the ADC clock speed of a 16 MHz Arduino is set to 125 kHz. Each conversion in AVR takes 13 clocks, and the sampling rate is 9615 Hz (125,000/13). The ATmega1280 board has 128 KB of flash memory for storing code. We use XBeeTM produced by MaxStream Inc. as the transceiver in our system. The outdoor range of XBeeTM is up to 100 m, and the radio frequency (RF) data rate is 250,000 bps (Digi 2014a). In order to put it on the Arduino board, we also need an Arduino XBee shield, which is right under the XBee (Fig. 9). The shield can be placed directly on the Arduino board and then the XBee can be embedded on the Arduino board via the shield so that Arduino can have the access to the wireless communication.

In our design, the data sending actions are event-triggered in order to reduce the cost of data communication. Once the tension sensor’s analog signals reach above 15 units (that is about 20 N), which means the athlete starts a throw, the sensor device will be able to start working and collect data automatically. By using the event-trigger, we resolved the issues of how to avoid receiving junk data.

Another critical idea used in our sensor node is an easy-release connector installed between the hammer and our system device. When the hammer is thrown away by the athlete, the connector will be released along with the hammer, which means the tension sensor cannot feel any tension at this time. Then the sensor node will stop collecting/transmitting data. During the athlete performs a movement, the sensor node keeps sending data to the receiver node in real-time. By applying the two important techniques, our system will be convenient and accurate for both coaches and athletes.

#### Receiver node

The receiver node is shown in Fig. 8. The receiver node consists of an XBee, which is the same type of the one used in the sensor node, and a laptop. The XBee in the receiver node also needs a shield, which is right under the XBee module (Fig. 9), to be connected to the computer via a USB cable. The end-user computer is used to receive, monitor and process the data sent from the sensor node.

### Programming and interface

In the sensor node, we can write codes in Arduino sketch which is a kind of software integrated development environment based on C/C++. Its library is related with AVR Libc and allows people to use its functions (Arduino 2014b). We can upload the program directly from the Arduino sketch to our Arduino Mega board so that we can control our sensor node and make an initial process when collecting and sending data. Our program was implemented based on the AnalogReadSerial (Arduino 2014c).

In the receiver node, we use MATLAB as our programming tool. We create a graphical user interface to show the data values of the two sensors in MATLAB. We also implement a program to process the data and plot the data. For minimizing electrical noise, the Butterworth filter is used when plotting the data. After the release (i.e. the sensor node was separated from the microcontroller), the collected data is sent to the receiver node, and it is filtered for a real-time plot in MATLAB. The cut-off frequency of the Butterworth filter in MATLAB is set to 0.2, which can provide a smooth and reasonable curve. The MATLAB GUI program is used to monitor and process real-time data on PC.

### System calibration

**Fig. 10:**
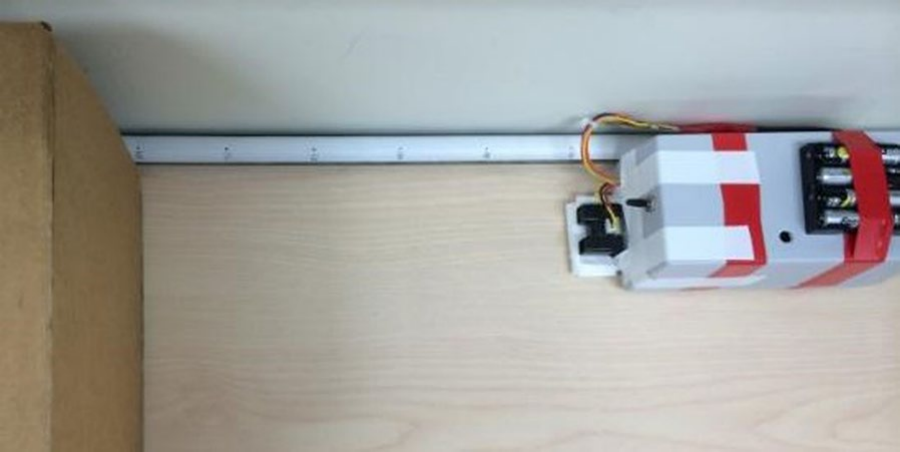
Calibration of the distance sensor

The last step before the application of the biofeedback device into practice is the system calibration, i.e. to convert the electrical output of sensors into the centimeter (distance sensor) or Newton (tension sensor). For both sensors, we finished the calibration tests in three different days to examine the reliability of our calibration. On each calibration day, we performed two sets of calibration test and the time for the calibration test on each day was different. For the distance sensor, one set of the calibration test consisted of 60, 70, 80, 90, 100, 110, 120, 130, 140 and 150 (Fig. 10). All calibration data was used for establishing interpolation equation (Eq. 2) for converting the electrical output into centimeters.

2 $$A = \frac{{ - 0.01579057581 \times O^{2} + 33.31143294 \times O - 319.9862609}}{O - 141.2605379},$$

where A is the distance in cm and O is the measured sensor electrical output. The residual sum of squares (RSS) was 2.488469061. The calibration results have revealed that the output of the sensor and the measured distance have a non-linear relationship and the error range of the interpolation Eq. 2 is acceptable (<5 mm).

**Fig. 11:**
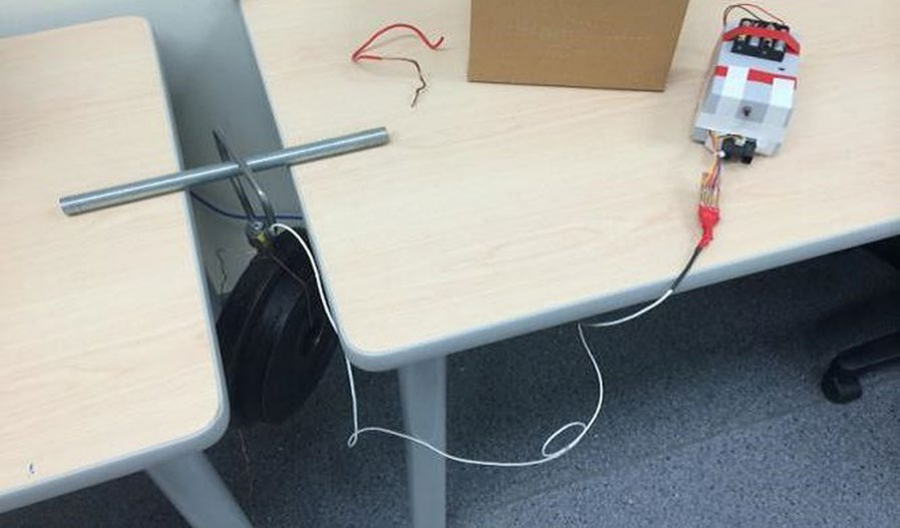
Calibration of the tension sensor

Figure 11 shows the process of the tension sensor calibration. Since the producer of the sensor has promised a high linearity within the measuring range (0–5000 N) (Omegadyne 2014), we have selected 4 standard weights for each set of calibration test: 4.545 kg (44.5 N), 6.818 kg (67.8 N), 9.090 kg (89.1 N) and 11.363 kg (111.4 N). The data of 3-day calibration was used to establish the linear equation (Eq. 3) for the conversion of the tension sensor’s electrical output.

3 $$A = 0.6031 \times O - 4.8064,$$

where A is the wire tension in N and O is the measured sensor electrical output. After Eq. 3 had been established, we verified the tension sensor by testing 13.636 kg (133.7 N), 15.909 kg (155.9 N), 18.181 kg (178.2 N) and 20.454 kg (200.5 N). The error is neglect-able (<1 N), which indicates a reliable linearity of the sensor.
